# Anodic dissolution mechanisms of iron in bentonite slurries

**DOI:** 10.1038/s41529-025-00681-9

**Published:** 2025-11-13

**Authors:** Pranav Vivek Kulkarni, Anna Igual-Munoz, Jean-Michel Sallese, Stefano Mischler

**Affiliations:** 1 EPFL–École Polytechnique Fédérale de Lausanne, Tribology and Interfacial Chemistry (TIC) Group, Lausanne, Switzerland; 2 EPFL–École Polytechnique Fédérale de Lausanne, STI GR-SCI-IEL, Lausanne, Switzerland

**Keywords:** Engineering, Materials science

## Abstract

The corrosion of iron or steel in contact with bentonite is a key factor affecting the long-term safety of radioactive waste disposal system. Previous studies focused on corrosion after long-term burial in compact bentonites, however, little work was dedicated to the corrosion of iron exposed to bentonite slurries, that can appear in case of fracture of the bentonite jacket separating steel from underground water. In this study, accelerated corrosion experiments were performed on pure iron in basic bentonite slurries (pH 9-10) using various electrochemical corrosion techniques. The anodic dissolution of iron was larger in more concentrated bentonite slurries and resulted in the formation of an acidic gel. This gel results from a cationic exchange between Fe^2+^ ions released by corrosion and protons from surface or edge locations in bentonite. Its growth appears to be governed by reactions at the gel-bentonite interface rather than diffusion processes.

## Introduction

Corrosion of iron and steel in contact with bentonite is a concern in nuclear waste disposal^1^. Disposal of radioactive waste in deep geological repositories (DGR) is the currently internationally accepted solution to isolate the radionuclides produced during nuclear fission from the biosphere. In most countries, the current plan is to encapsulate the spent nuclear fuel and high-level waste in metallic disposal canisters, which will be emplaced deep underground in a geological repository whose tunnels will then be backfilled with bentonite clay. This requires canisters with lifetimes usually between 1000 and 100,000 years, while safety assessment for the repositories is typically done for 1,000,000 years^2–6^. The corrosion of canister materials in bentonite under conditions expected in a repository in the long-term (anoxic, saturated with rock porewater, background rock temperature) is being extensively studied in various national programmes^7,8^ and is nowadays well understood^9^. However, during the repository evolution, localized or short-lived heterogeneities in bentonite saturation may occur^10,11^ leading to exposure of iron to bentonite slurries. Same exposure could occur in case of groundwater ingress via large-scale cracks. Although such a situation is considered in DGR as a conservative^12^ scenario^13^ (due to the swelling and sealing properties of compacted bentonite), Fe interactions with bentonite slurries need to be understood in case the above-mentioned events take place.

Corrosion of low-carbon steel in bentonite slurries was investigated by Wei et al.^11^. They measured potentiodynamic polarization curves in a Na_2_SO_4_ + NaHCO_3_ + NaCl solution with 0 to 33% weight of dissolved bentonite. They observed that the increasing bentonite content decreases the cathodic reactions mainly due to limitations to the oxygen diffusion. The bentonite content also increases the anodic dissolution of steel. Long-term experiments (up to 120 days) at the corrosion potential show after 16 days immersion the appearance of a rust layer followed by the coagulation of bentonite from the suspension. Those authors^11^ argued, based on impedance data, that the formed layers protect the steel against corrosion by inhibiting the oxygen access to the corroding surface.

Researchers in ref. ^14^ observed, based on potentiodynamic polarization curves, that steel was pseudo-passive in a 0.05 M NaHCO_3_ solution but underwent more severe active dissolution in presence of bentonite.

Moreover, it has been reported that, the corrosion of iron is higher in water saturated compacted bentonite as well as in bentonite slurries compared to bulk solutions mimicking porewater chemistry^15^.

Kitayama (2020)^16^ carried out electrochemical corrosion studies of the steel with compacted buffer material (bentonite-sand mixtures) saturated with basic (pH>8.5) synthetic seawater solutions, moreover, compared the results without the buffer materials. They found that passivation of steel was prevented in the presence of compacted bentonite buffer materials even at the high pH ( > 10) environments, in contrary to the conditions without buffer materials. Taniguchi^17^ et al. have made the very similar observations regarding the passivation in compacted bentonite and the carbonate environments. However, the mechanisms by which it occurs are relatively unexplored.

From this literature analysis, one may conclude that discrepancies exist about the effect of bentonite on the corrosion of iron in bentonite slurries or water saturated compacted buffer materials. In certain cases, bentonite is found inhibiting the corrosion by forming mixed rust/bentonite layers. In other cases, the detrimental effect of bentonite on corrosion was attributed to the suppression of iron passivity.

This paper aims at investigating the electrochemical behavior of iron in the presence of bentonite slurries and identifying the involved corrosion mechanisms. For this, potentiodynamic, potentiostatic and galvanostatic experiments involving polarization of iron electrodes in bentonite/water slurries of different concentrations will be carried out. The corrosion products will be characterised gravimetrically and using EDS chemical analysis.

## Results

### OCP and LPR

**Fig. 1:**
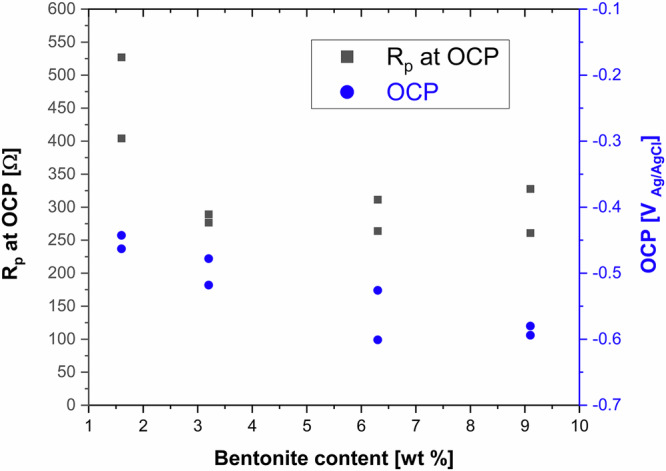
OCP and the LPR (R_p_) in bentonite slurries. The OCP and LPR measured after 60 s immersion in bentonite slurries of different concentrations: black square points: R_p_ on the left ordinate, blue circle points: OCP values on the right ordinate.

During the first 60 s immersion OCP decreased cathodically with maximum changes limited to 100 mV. The LPR measurement were characterised by a nearly perfect linear relation between potential and current without any distortion typically associated to capacitive effects due to passive films or other corrosion products. Figure 1 shows the OCP values and of polarization resistance-R_p_ with bentonite concentration. While the OCP steadily becomes more cathodic with bentonite content, the R_p_ values exhibit a decrease when passing from 1.6% to 3.2% bentonite but remain constant independently on further increase in bentonite content.

### Potentiodynamic polarization

**Fig. 2:**
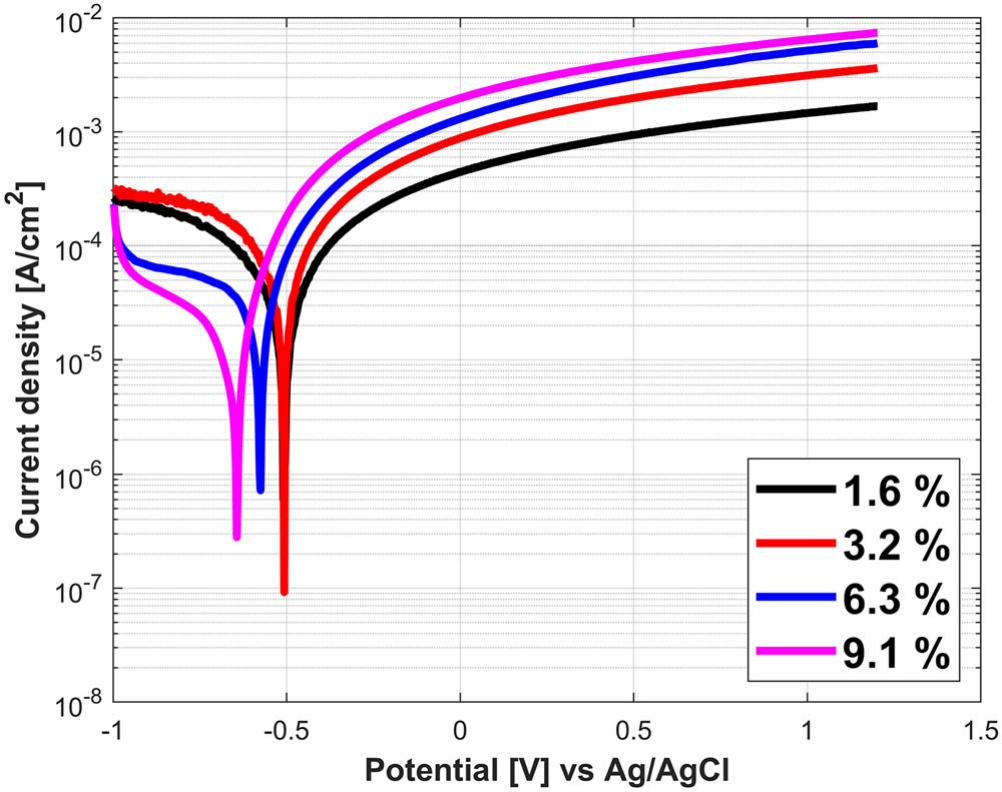
Potentiodynamic polarization curves of iron in bentonite slurries. Potentiodynamic scans for different bentonite slurries:1.6% slurry (black line), 3.2% slurry (red line), 6.3% slurry (blue line), 9.1% slurry (magenta line).

Typical potentiodynamic polarization curves of the iron samples in different bentonite slurries are shown in Fig. 2. The cathodic domain lies between −1 V_Ag/AgCl_ to the E_corr_ (~ −0.5 V_Ag/AgCl_), while the active domain starts from the E_corr_ and higher potentials. As the bentonite concentration increases, the absolute cathodic current densities and the corrosion potentials E_corr_ decrease while the anodic current densities show a opposite trend i.e., increasing with higher bentonite concentrations. These results are in good agreement with previously published observations^11^. The difference in cathodic current densities could be explained by a lower oxygen concentration and decreased oxygen transport linked with the higher salinity and viscosity of concentrated bentonite slurries^11^.

**Fig. 3:**
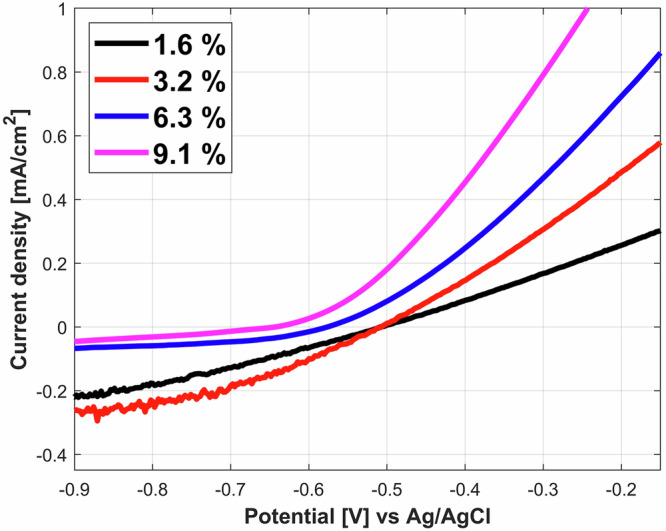
Polarization curves of iron in bentonite slurries in a linear scale. Linear scale plot of potentiodynamic polarization scans for different bentonite slurries: 1.6% slurry (black line), 3.2% slurry (red line), 6.3% slurry (blue line), 9.1% slurry (magenta line).

The polarization curves in the vicinity of E_corr_ are shown in Fig. 3 using linear scales. The polarization curve measured in the most diluted slurry (1.6%) has a linear shape indicating an ohmic control of the kinetics likely due to the low conductivity (due to low salinity) of the solution. For the other concentrations, the observed exponential relation between potential and current clearly indicates that the anodic reaction is under charge transfer control. However, above a potential of −0.4 V a linear behaviour appears again for all bentonite concentrations suggesting that the limited conductivity of the slurries starts controlling the anodic reaction. Interestingly, the highest concentrated bentonite slurries, thus with the highest salinity, exhibit highest slopes in the linear part of Fig. 3, corresponding to lower resistance or higher conductivity.

The samples extracted from the slurries after completing the polarization curves were found covered by greyish corrosion products. The samples polarised in the 6.3 and 9.1 wt% slurries exhibited full coverage by this corrosion product.

### Effect of bentonite content on the potentiostatic anodic dissolution

**Fig. 4:**
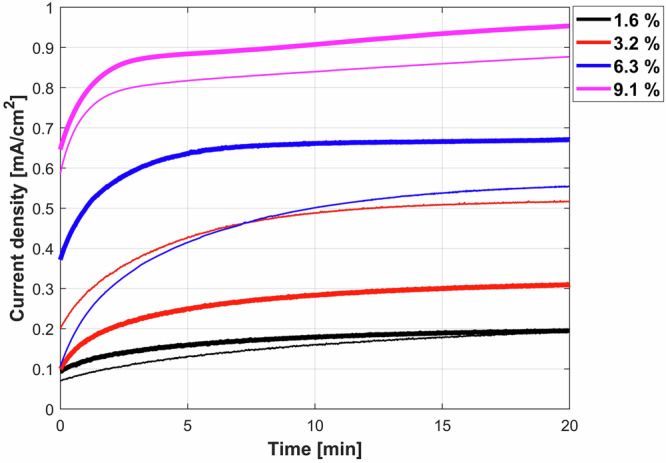
Anodic potentiostatic current evolution of iron in different bentonite slurries. Evolution of the current densities measured during anodic potentiostatic tests (E_applied_ = −0.4 V_Ag/AgCl_) for 20 mins, in bentonite slurries of different concentrations: 1.6% slurry (black line), 3.2% slurry (red line), 6.3% slurry (blue line), 9.1% slurry (magenta line). Fine lines indicate the replications.

Figure 4 presents the evolution of the current densities measured during anodic potentiostatic tests (E_applied_ = −0.4 V_Ag/AgCl_) in bentonite slurries of different concentration. For the initial 5 mins, continuously rising current densities were observed, which apparently reached constant values over the next period of the measurement. Particularly, slurries with higher bentonite contents show higher current densities, indicative of increased iron dissolution at the given anodic potential.

**Fig. 5:**
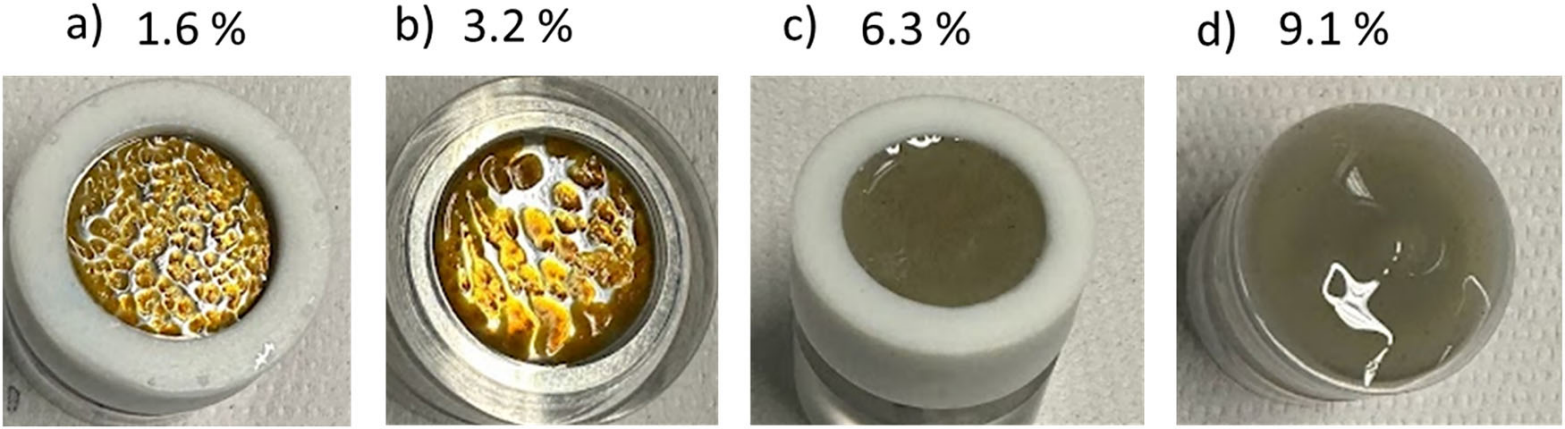
Iron surfaces after potentiostatic tests. Representative images of the iron surfaces at the end of the potentiostatic tests carried out for 20 mins in different bentonite slurries (**a**—1.6%, **b**—3.2%, **c**—6.3%, **d**—9.1%).

The surface condition of the iron samples was checked after completion of the potentiostatic experiments and shown in Fig. 5. The surfaces of the samples were found covered with a millimetre range thick gel like corrosion product. Depending on bentonite content, the gel partially (1.6 and 3.2 wt%) or completely (6.3 and 9.1 wt%) covered the iron surface. In case of low bentonite content (1.6 and 3.2 wt%) some reddish corrosion products were also observed. The presence of corrosion products is consistent with the observed weight increase of the electrodes upon anodic polarization (see Table 1).

Table 1 summarises the parameters in terms of solution resistance (R_s_), pH of the solution, mass change of the iron electrode, properties of the gel (pH and weight of the gel), and anodic charge (determined by integrating the current over the whole polarization time) during to 20 minutes of the anodic potentiostatic tests in Fig. 4.

**Table 1:** Parameters derived from the anodic potentiostatic tests for different bentonite slurries carried out for 20 mins

Bentonite content in the slurry, wt %	R_s -start_, Ω at t = 0 min	R_s- finish_, Ω at t = 20 min	Gel pH	Slurry pH	Mass change, g	Q (Integrated charge), C
1.6	415	403	5.5 ± 0.3	9.5 ± 0.5	0.16	0.51
	468	457	n.d.	8.7 ± 0.3	0.13	0.58
3.2	163	157	6.5 ± 0.5	9.5 ± 0.5	0.23	1.54
	278	268	6.4 ± 0.4	9.0 ± 0.5	0.11	0.91
6.3	141	136	6.5 ± 0.5	9.5 ± 0.5	0.45	1.56
	118	111	5.8 ± 0.3	9.5 ± 0.5	0.50	2.16
9.1	94	81	5.5 ± 0.3	10 ± 0.5	1.72	2.82
	88	75	6.5 ± 0.5	10 ± 0.5	1.82	3.05

“R_s-start_” and “R_s-finish_” represent the solution resistance at the beginning (t = 0 min) and at the end (t = 20 min) of the potentiostatic tests, respectively.

### Effect of time on the potentiostatic anodic dissolution of iron in bentonite slurry

**Fig. 6:**
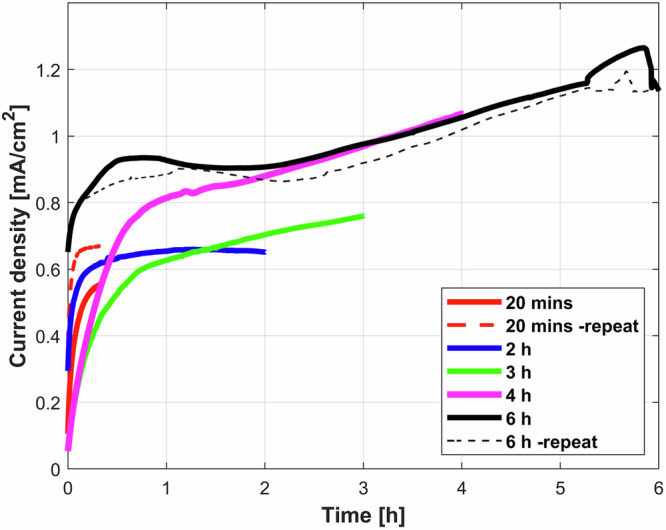
Temporal evolution of current densities during the anodic potentiostatic tests. Evolution of the current densities measured during anodic potentiostatic tests (E_applied_ = −0.4 V_Ag/AgCl_) in 6.3% wt bentonite slurry for different times: 20 mins (red lines), 2 h (blue line), 3 h (green line), 4 h (magenta line), 6 h (black lines). Dashed lines indicate the replications.

Anodic potentiostatic tests for different times (20 mins to 6 hours) were carried out to study the kinetics of growth of the gel in a specific electrolyte, the 6.3% slurry. Figure 6 shows the current density evolutions for the anodic potentiostatic test at −0.4 V_Ag/AgCl_ for different times in the 6.3 wt% bentonite slurry. The current densities show a steep rise for initial 10–15 mins, and after a while show a steady rise with time. Again, a greyish gel (like the one shown in Fig. 5 for high bentonite contents) was observed covering the entire surface formed on the iron electrode upon polarization (with ~1 cm range at high polarization times, i.e., 6 h).

Table 2 summarizes the extracted parameters in terms of solution resistance, properties of the gel (pH and weight) and anodic charge resultant from the potentiostatic test from Fig. 6.

**Table 2:** Parameters derived from the anodic potentiostatic tests for 6.3% wt bentonite slurry carried out as a function of time

Polarization time	R_s-start_, Ω	R_s-finish_, Ω	Gel pH	Slurry pH	Mass change, g	Q (Integrated charge), C
2 h	130	111	5.0 ± 0.5	9.5 ± 0.5	1.56	12.93
3 h	138	88	5.0 ± 0.5	9.5 ± 0.5	2.09	19.13
4 h	104	62	5.0 ± 0.5	9.5 ± 0.5	2.44	34.52
6 h	100	60	5.5 ± 0.5	9.5 ± 0.5	3.85	61.64
	98	60	5.5 ± 0.5	9.5 ± 0.5	3.50	57.92

### Galvanostatic experiments

**Fig. 7:**
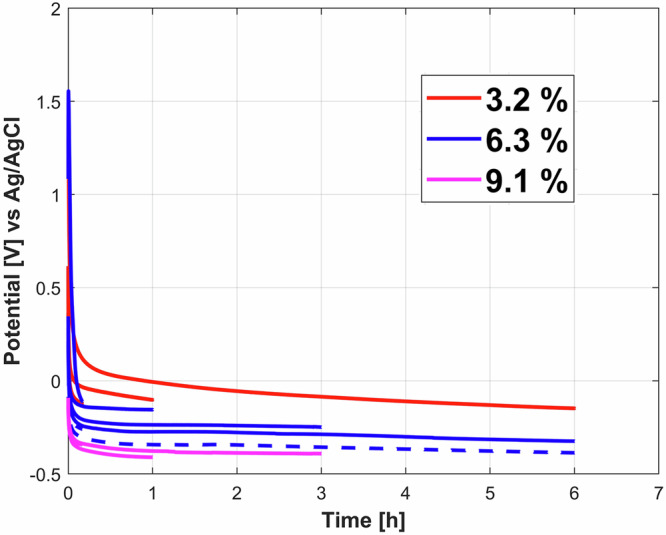
Potential temporal evolution for the anodic galvanostatic tests. Potential evolutions for applied anodic current density of 0.7 mA/cm^2^ for different times (0.16 to 6 h) and different bentonite slurries: 3.2% slurry (red line), 6.3% slurry (blue line), 9.1% slurry (magenta line). Repetitions are shown with dashed curves.

Potential evolution with time of the galvanostatic experiments carried out in the different bentonite slurries at current densities of 0.7 mA/cm^2^ (2 mA current) are shown in Fig. 7. Different polarization times were considered to assess the time dependency on the iron dissolution. Lower bentonite content i.e., 3.2% slurries showed more positive/anodic potentials, followed by 6.3% slurries while the highest bentonite content slurries showed more negative potential evolutions. For all the slurries potentials show a sharp decrease during the initial 10–15 mins, followed by a steady evolution with time.

Table 3 summarizes the extracted parameters in terms of properties of the gel (pH and mass change) for galvanostatic tests (Fig. 7). Anodic charge is calculated by multiplying the galvanostatic current (2 mA) by the galvanostatic experimental time in seconds.

**Table 3:** Parameters derived from the anodic galvanostatic tests for different bentonite slurries carried out as a function of time

Bentonite content in the slurry, wt %	Polarization time	Gel pH	Slurry pH	Mass change, g	Q (Integrated charge), C
3.2	0.16 h	5.5 ± 0.3	9 ± 0.5	0.31	1.2
	1 h	5.5 ± 0.3	9 ± 0.5	0.76	7.2
	6 h	5 ± 0.3	9 ± 0.5	1.53	43.2
6.3	0.16 h	7 ± 1	9.5 ± 0.5	0.35	1.2
		5 ± 1	9.5 ± 0.5	0.44	1.2
	1 h	5.5 ± 0.3	9.5 ± 0.5	0.87	7.2
	3 h	5.5 ± 0.5	9.5 ± 0.5	1.84	21.6
	6 h	5 ± 0.5	9.5 ± 0.5	3.65	43.2
		5 ± 0.5	9.5 ± 0.5	3.84	43.2
9.1	0.16 h	7 ± 1	10 ± 0.5	0.73	1.2
	1 h	6.5 ± 0.5	10 ± 0.5	2.53	7.2
	1 h	6.5 ± 0.5	10 ± 0.5	2.22	7.2
	3 h	6.5 ± 0.5	10 ± 0.5	9.01	21.6

### EDS analysis of the dried gel

Table 4 shows the EDS elemental composition (4-point average, coated with 5 nm gold layer) of the vacuum dried gel from a product formed during 6 h galvanostatic test in 6.3% slurry and of the MX-80 bentonite (4-point average of a dried slurry droplet, coated with 5 nm of gold layer) for comparison. The analysis reveals that the gel contained iron, up to 5 atomic percent (at. %). It is also important to note that the bentonite clay contains up to ~2 at. % Fe. Therefore, there is incorporation of dissolving Fe in the gel.

**Table 4:** EDS-elemental composition of the gel and MX-80 bentonite in atomic percentage

Gel composition [3- EDS point average], at. %	At%	Fe	O	Na	Mg	Al	Si	K	Ca	S
	Average	5.2	67.9	-	0.9	7.4	18.4	-	-	0.3
	Std. Dev.	0.3	0.5	-	0	0.2	0.7	-	-	0
MX-80 Bentonite composition, at. %	Average	2.2	62.4	1.5	1.1	9.3	23.2	0.2	0.2	-
	Std. Dev.	0.3	1.4	0.1	0.1	0.4	1.3	0.1	0	-

## Discussion

First, for ‘the gel formation reactions’—upon the introduction of bentonite ([BN]) clay into pure water, ion exchange occurs, wherein the sodium ions from the bentonite are replaced by protons derived from water hydrolysis, this has been well-reported in the literature^18^ with Eq. (1). This exchange process results in the accumulation of hydroxyl ions within the slurry, imparting a basic character to the solution, as evidenced by the pH levels observed in this study, ranging from 9 to 10.

1 $${Na}{\left[{BN}\right]}^{\left(-q\right)}+\,{H}_{2}O\,\to {\rm{H}}{\left[{BN}\right]}^{\left(-q\right)}\,+\,O{H}^{-}+\,{{Na}}^{+\,}$$

In addition to this, partial dissolution of calcite and some accessary minerals also contribute to the alkalinity of the slurry.

In the present experiments, the continuous dissolution of iron combined with the negative charge of suspended bentonite particles, might lead to a discharge mechanism (BN charge from (-q) to (-q + 1) as described in Eq. (2)) and already reported in the literature^19–21^.

2 $${\rm{H}}{\left[{BN}\right]}^{\left(-q\right)}+{{Fe}}^{2+}\to {Fe}{\left[{BN}\right]}^{\left(-q+1\right)}+{H}^{+}$$

The consequences of the acidification associated to bentonite discharge (Eq. (2)) can lead to different association modes of bentonite particles some of which (face to face and edge to edge) generate band-like and card-house structures resulting in gel formation^22^. This proton release primarily occurs from the surface hydroxyl groups located on the bentonite mineral particles, mainly at the edge sites of montmorillonite, which is the prime component of bentonite. These edges are proton-active sites and are involved in surface complexation reactions with Fe^2+^ from dissolution. The interaction of Fe²⁺ with these sites leads to the displacement and release of protons into the solution^20,21,23^. This supports the hypothesis that the gel formation observed in this study was induced by Fe^2+^ / H^+^ (Eq. (2)) exchange in the suspended bentonite and subsequent slurry acidification. In addition to these exchanges, hydrolysis of Fe^2+^ ions at the interface may also contribute to the acidification, although its contribution could be minor.

Next, for ‘the growth mechanisms of gel’—the mass of the gel M_gel_ can be derived from the weight difference M_diff_ of the sample after and before polarization by assuming that the gel is the only corrosion product and that the release of iron ions into the bentonite slurry is negligible. Reddish corrosion products (probably rust) were observed only for short polarization times in diluted slurries Fig. 5, but not when thick gels were covering the electrodes. Moreover, the mass of oxidized iron, as calculated from the electrical charge passed during the experiments (integral of current over time) and converted into mass using Faraday’s law (oxidation valence of 2) is very small and less than 1% of the measured M_diff_ values. Thus, a possible iron release into the slurry is negligible. According to the above considerations, the mass of the gel M_gel_ can approximately considered corresponding to the measured weight difference M_diff_.

**Fig. 8:**
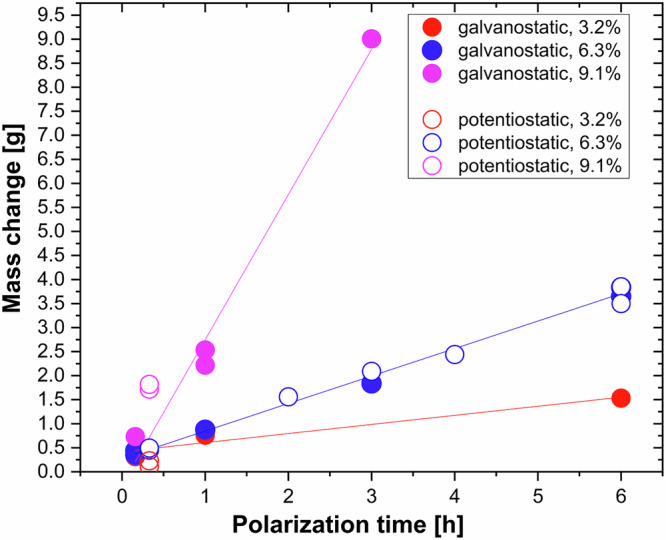
Mass change with polarization time. Change in electrode mass for tests conducted under potentiostatic (hollow dots) and galvanostatic (solid dots) conditions in different bentonite slurries:3.2% (red), 6.3% (blue) and 9.1 wt. % (magenta).

The evolution of the gel mass with polarization time for tests conducted under potentiostatic and galvanostatic conditions in the 3.2%, 6.3% and 9.1% slurries is shown in Fig. 8.

Interestingly, the growth rate of the gel remains constant over the entire investigated time interval. This indicates that the gel formation limiting step is not diffusion of iron or hydronium ions through the gel, since, in this case, one would have expected a square root relation between mass of the gel and polarization time. More likely, the limiting step is exchange reaction (2) of Fe^2+^ to H^+^ occurring at the gel-slurry interface by reaction of suspended bentonite particles with iron ions from the gel. This heterogeneous reaction appears to be limited by the access of bentonite particles to the interface. Indeed, higher bentonite concentrations yield higher gel growth rate (slope of curves in Fig. 8).

Regarding ‘the gel formation and corrosion of iron’—the formation of the gel upon release of Fe^2+^ ions into the slurry maintains iron in contact with an acidic environment which prevents its passivation and promotes its corrosion. This may have important practical consequences for DGRs in case of bentonite cracking accompanied by groundwater intake. In such a situation, bentonite slurries may develop and significantly enhance local (at the scale of bentonite cracks) corrosion of steel canisters. However, in the extrapolation of the results obtained in this work to real applications, one must consider following two factors.

The first one, is related to the minimum amount of iron that need to be dissolved to initiate the gel formation. Indeed, the gel formation requires lowering the pH below the isoelectric point of bentonite which is around pH 7^22^ to minimize electrostatic repulsion forces between suspended bentonite particles. According to Eq. (2) a minimum amount of ferrous iron ions is necessary to release enough proton to lower the pH below 7 and thus trigger gel formation. In the present experiments, the imposed electrochemical conditions imply high currents and hence high release rates of iron probably more than sufficient to trigger gel formation. In real situations, the canister steel is in principle not polarized and so the resulting corrosion rates are much lower than in the present experiment and possibly below the critical threshold for gel formation. Electrochemical experiments conducted at lower current densities could reveal the threshold corrosion rate necessary to initiate gel formation. Furthermore, during Fe corrosion concurrent hydroxyl ions may be produced cathodically in the near vicinity, and together with HCO^3-^ (the groundwater ions) could neutralize the resulting acidification.

A second point to be considered is that ground water contains cations that could interfere^10^ with the exchange mechanisms described in Eq. 2 and thus influence the acidification and the gel formation. Indeed, it is known that ions, such as Ca^2+^ or Na^+^, affects^18^ the intake of protons by bentonite (Eq. (1)) and may compete with Fe^2+^ ion in the incorporation in bentonite (Eq. (2)). Lastly, additional oxidation of Fe^2+^ to Fe^3+^ due to oxygen could provide an additional Fe-lowering mechanism.

In summary, the study on the anodic dissolution of iron in bentonite slurry has led to the following conclusions.

- Increasing bentonite contents in the water suspensions were found to inhibit the cathodic reduction of oxygen and promote the anodic dissolution of iron.
- The anodic iron dissolution process results in the formation of a gel-like substance in presence of bentonite slurries. This gel is composed of bentonite clay, incorporating approximately 3 atomic percent of dissolved iron.
- The gel is found to be acidic in nature with a typical pH of 5 to 6.5 ± 0.5.
- The mechanism behind this gel formation appears to be closely related to the ion exchange process between the dissolved iron ions and the protons in the bentonite.
- The acidic gel covering the iron surface explains the detrimental effect on anodic dissolution of iron by bentonite and is most likely responsible for the suppression of passivity.

## Methods

### Sample preparation

The electrochemical behavior of pure iron (99.8 weight percent purity, Goodfellow) was considered. The utilized iron was found to have some inclusions of the types Mn-Si, Mn-S and Si-O. For preparation, iron samples were first cut in form of discs (⌀ 24 mm, thickness 3 mm) and then polished (one flat side) with a semi-automatic polishing machine (Buhler) with different grids of SiC papers (P-220, P-1000, P-2400, P-4000) for 5 mins each at 150 N force for 6 samples, and finally lap-polished with ¼ um diamond suspension for 3 mins. The samples were rinsed with water after every step. After the diamond polishing, the final rinsing was done with ethanol. Thereafter, samples were dried with air and stored in a vacuum before use. The average surface roughness (Ra) of the used pure iron samples was 20 ± 3 nm and was reproducible in the other polished samples.

### Bentonite slurries

The MX-80 Na- Wyoming bentonite (same as in the field emplacement experiment, refer to ref. ^24^) was used in the study. This bentonite contains several key minerals, according to detailed mineralogical analysis of the granular bentonite carried out in ref. ^25^: Na-Smectite: 79.8 ( ± 3.0)%, Quartz: 7.0 ( ± 0.3)%, K-Feldspar: 4.0 ( ± 0.4)%, Illite: 3.2 ( ± 0.6)%, Na-plagioclase: 3.0 ( ± 0.5)%, Calcite: 1.2 ( ± 0.2)%, Gypsum (n.d.), Cristobalite: 1.1 ( ± 0.6)%, Pyrite: 0.3 ( ± 0.1)% and Siderite: 0.3 ( ± 0.2)%. Different proportions of this bentonite- 5 g, 10 g, 20 g and 30 g were added to the 300 mL of distilled water to obtain slurries with bentonite concentrations of 1.6, 3.2, 6.3 and 9.1 wt%, respectively. During mixing, the mixture was constantly stirred to get fluid slurries. The mixtures were kept still for homogenization for at least 12 h before using them as an electrolyte. All the slurries were used for the electrochemical experiments within a week after their preparation. The pH of all the slurries was found to be in a range of 9.5 ( ± 0.5), as always checked before the electrochemical experiments.

### Electrochemical methods

A classical three-electrode set-up was used for the electrochemical experiments. The reference electrode was an Ag/AgCl electrode (3 M KCl, 0.205 V with respect to the standard hydrogen electrode, SHE) and a graphite rod was used as a counter electrode. Iron discs, prepared according to the procedure described in ‘sample preparation’, were mounted on a special holder exposing to the solution a polished iron area of 2.83 cm^2^. In the holder, the backside of the iron disk was electrically connected to the potentiostat as working electrode. The Autolab PGSTAT302N potentiostat (Metrohm) was used for carrying out the electrochemical measurements. During experiments the solution was constantly exposed to air and stirred using a magnetic stirrer operating under identical conditions for all experiments.

For—open circuit potential, linear polarization resistance and potentiodynamic polarizations, two tests under the same conditions were systematically performed for checking the reproducibility of the measurements. The procedure involved following sequential steps:

- Open Circuit Potential (OCP) was recorded for 60 seconds.
- Linear Polarization Resistance (LPR) was measured by imposing a potential variation of ±20 mV around the OCP, with a scan rate of 2 mV/s. The LPR value was derived from the current-potential curve as a slope within its linear segment.
- Potentiodynamic polarization measurements were conducted, ranging from a cathodic potential of −1 V_Ag/AgCl_ to an anodic potential of +1.2 V_Ag/AgCl_, at a scan rate of 2 mV/s.

For anodic potentiostatic experiments, tests in the different slurries were carried at the anodic potential of −0.4 V_Ag/AgCl_ for 20 mins. For time-evolution study, a set of potentiostatic tests were carried out in the 6.3% slurry for times of 20 mins to 6 hours. Electrochemical Impedance Spectroscopy (EIS) measurements were performed under the selected applied potential at the beginning (t = 0) and at the end of the potentiostatic tests by applying an anodic perturbation of ±10 mV to the applied voltage, across a frequency spectrum from 10^5^ to 100 Hz. These measurements were carried out to determine the solution resistance in between the working and the reference electrode. For this, the impedance value obtained at 10^4^ Hz, i.e., in the middle of the frequency domain where the phase shift was ~zero, was considered as the solution resistance.

For galvanostatic experiments, tests were conducted using the same set-up for different bentonite slurries but by imposing, instead of a potential, a constant current of 2 mA (0.7 mA/cm^2^). No impedance was measured under galvanostatic conditions.

The mass of the iron specimens was measured before and at the end of the experiments in order to quantify the amount of corrosion and formed corrosion products. The pH values of the corrosion products adhering to the iron electrode and of the slurries, after the anodic potentiostatic and galvanostatic tests, were determined using pH test strips with precision of ±0.3 to ±0.5 upon completion of the experiments. The pH strips were immersed—inserted down in the corrosion product (gel like) at some distance.

The surface analysis such as morphology and the chemical analysis of the corrosion products were carried out with the scanning electron microscope (SEM) and energy dispersive x-ray spectrometer (EDS) with SEM-Gemini (Zeiss made). The SEM imaging was carried out with a 3 kV, ~200 pA electron beam while the EDS acquisitions was carried out at 10 kV, 2000 pA primary beam.

## Supplementary information

Supplementary Information

## Data Availability

Data are provided within the manuscript.

## References

[1] Milodowski, A. E. et al. Svensk Kärnbränslehantering AB Mineralogical investigations of the interaction between iron corrosion products and bentonite from the NF-PRO Experiments (Phase 1). (2009).

[2] Patel, R. et al. *Canister Design Concepts for Disposal of Spent Fuel and High Level Waste*. www.nagra.ch (2012).

[3] https://www.ensi.ch/en/2009/11/11/g03-specific-design-principles-for-deep-geological-repositories-and-requirements-for-the-safety-case/ (2009). *Guidelines in Force | G03 Specific Design Principles for Deep Geo-Logical Repositories and Requirements for the Safety Case-ENSI-G03/e*.

[4] Kärnbränslehantering, S. A. *Svensk Kärnbränslehantering AB Corrosion Calculations Report for the Safety Assessment SR-Site*. www.skb.se (2011).

[5] Scully, J. R. & Edwards, M. *Review of the NWMO Copper Corrosion Allowance*. www.nwmo.ca (2013).

[6] Scully, J. R., Féron, D. & Hänninen, H. *Review of the NWMO Copper Corrosion Program*. www.nwmo.ca (2016).

[7] Muñoz, A. G. et al. WP15 ConCorD state-of-the-art report (container corrosion under disposal conditions). *Front. Nuclear Eng.* **3**, (2024).

[8] Wersin, P. et al. Unravelling the corrosion processes at steel/bentonite interfaces in in situ tests. *Mater. Corros.* **74**, 1716–1727 (2023).

[9] Padovani, C. et al. The corrosion behaviour of candidate container materials for the disposal of high-level waste and spent fuel–a summary of the state of the art and opportunities for synergies in future R&D. *Corros. Eng. Sci. Technol.* **52**, 227–231 (2017).

[10] Kärnbränslehantering, S. A. & Liu, J. *R-06-103 Physical and Chemical Stability of the Bentonite Buffer*. *CM Gruppen AB* www.skb.se (2007).

[11] Wei, X. et al. Effects of bentonite content on the corrosion evolution of low carbon steel in simulated geological disposal environment. *J. Mater. Sci. Technol.* **66**, 46–56 (2021).

[12] Braithwaite, L. et al. Galvanic Coupling of Copper and Carbon Steel in the Presence of Bentonite Clay and Chloride. *J. Electrochem Soc.* **169**, 051502 (2022).

[13] Ning, Z., Zhou, Q., Li, N., Macdonald, D. D. & Zhou, J. Understanding copper corrosion in bentonite-enriched environments: Insights from the point defect model. *Electrochim Acta* **505**, (2024).

[14] Wei, X., Dong, J. & Ke, W. Progress on a corrosion study of low carbon steel for HLW container in a simulated geological disposal environment in China. *Corros. Commun* **1**, 10–17 10.1016/j.corcom.2021.05.002 (2021).

[15] King, F. *Technical Report 08-12, Corrosion of Carbon Steel under Anaerobic Conditions in a Repository for SF and HLW in Opalinus Clay*. https://nagra.ch/en/downloads/technical-report-ntb-08-12-2/ (2008).

[16] Kitayama, A., Taniguchi, N. & Mitsui, S. Electrochemical behavior of carbon steel with bentonite/sand in saline environment. *Mater. Corros.* **72**, 211–217 (2021).

[17] Taniguchi, N., Honda, A. & Ishikawa, H. Experimental Investigation of Passivation Behavior and Corrosion Rate of Carbon Steel in Compacted Bentonite. *MRS Proc.* **506**, 495 (1997).

[18] Kaufhold, S., Dohrmann, R., Koch, D. & Houben, G. The pH of aqueous bentonite suspensions. *Clays Clay Min.* **56**, 338–343 (2008).

[19] King, F., Kolář, M. & Keech, P. G. Simulations of long-term anaerobic corrosion of carbon steel containers in Canadian deep geological repository. *Corros. Eng. Sci. Technol.* **49**, 456–459 (2014).

[20] Kiczka, M. *et al*. Reactive transport modelling of iron bentonite interaction in the FEBEX in situ experiment. *Applied Geochemistry* **170**, (2024).

[21] Bradbury, M. H. & Baeyens, B. *Contaminant Hydrology A Mechanistic Description of Ni and Zn Sorption on Na-Montmorillonite Part II: Modelling*. *Baeyens / Journal of Contaminant Hydrology* vol. 27 (1997).

[22] Choo, K. Y. & Bai, K. Effects of bentonite concentration and solution pH on the rheological properties and long-term stabilities of bentonite suspensions. *Appl Clay Sci.* **108**, 182–190 (2015).

[23] Hunter, F., Bate, F., Heath, T., Hoch Serco Assurance, A. & Kärnbränslehantering, S. A. Geochemical investigation of iron transport into bentonite as steel corrodes. (2007).

[24] Müller, H. R. et al. Implementation of the full-scale emplacement (FE) experiment at the Mont Terri rock laboratory. *Swiss J. Geosci.* **110**, 287–306 (2017).

[25] Garitte, B. et al. *Arbeitsbericht NAB 15-24 FE/LUCOEX: Requirements, Manufacturing and QC of the Buffer Components*. www.nagra.ch (2015).

