# Supplementary material for: Anodic dissolution mechanisms of iron in bentonite slurries
Source: Npj Mater Degrad. 2025 Nov 13;9(1):137. doi: 10.1038/s41529-025-00681-9 (PMC12615252; doi:10.1038/s41529-025-00681-9)
Supplement: Supplementary file 1 — Supplementary Information [file 41529_2025_681_MOESM1_ESM.pdf]

Supplementary Information (S.I.) for:

Anodic dissolution mechanisms of iron in bentonite slurries

Pranav Vivek Kulkarni[1], Anna Igual-Munoz[1], Jean-Michel Sallese[2], Stefano Mischler[1]

1) EPFL–École Polytechnique Fédérale de Lausanne, Tribology and Interfacial Chemistry (TIC) Group, Switzerland

2) EPFL–École Polytechnique Fédérale de Lausanne, STI GR-SCI-IEL, Switzerland

Supplementary Information:

<u>Titration with Fe(II) salts with bentonite slurry, procedure and results:</u>

Laboratory grade Fe(II) salts namely, $FeCl_2.4H_2O$ and $FeSO_4.7H_2O$, were added to the 100 mL of 6.3% bentonite slurry samples in varied quantities (Table S1 and Table S2). Slurry was made using the procedure detailed in method section of the main manuscript.

The pH (Figure S1) and visual consistency (Figure S2) of the resulting baths were noted.

Figure S1 reports the slurry pH after addition of Fe(II) salts. Table S1 and Table S2 detail the amounts of salts added and the pH information. Figure S2 notes the slurry consistency after Fe(II) addition.

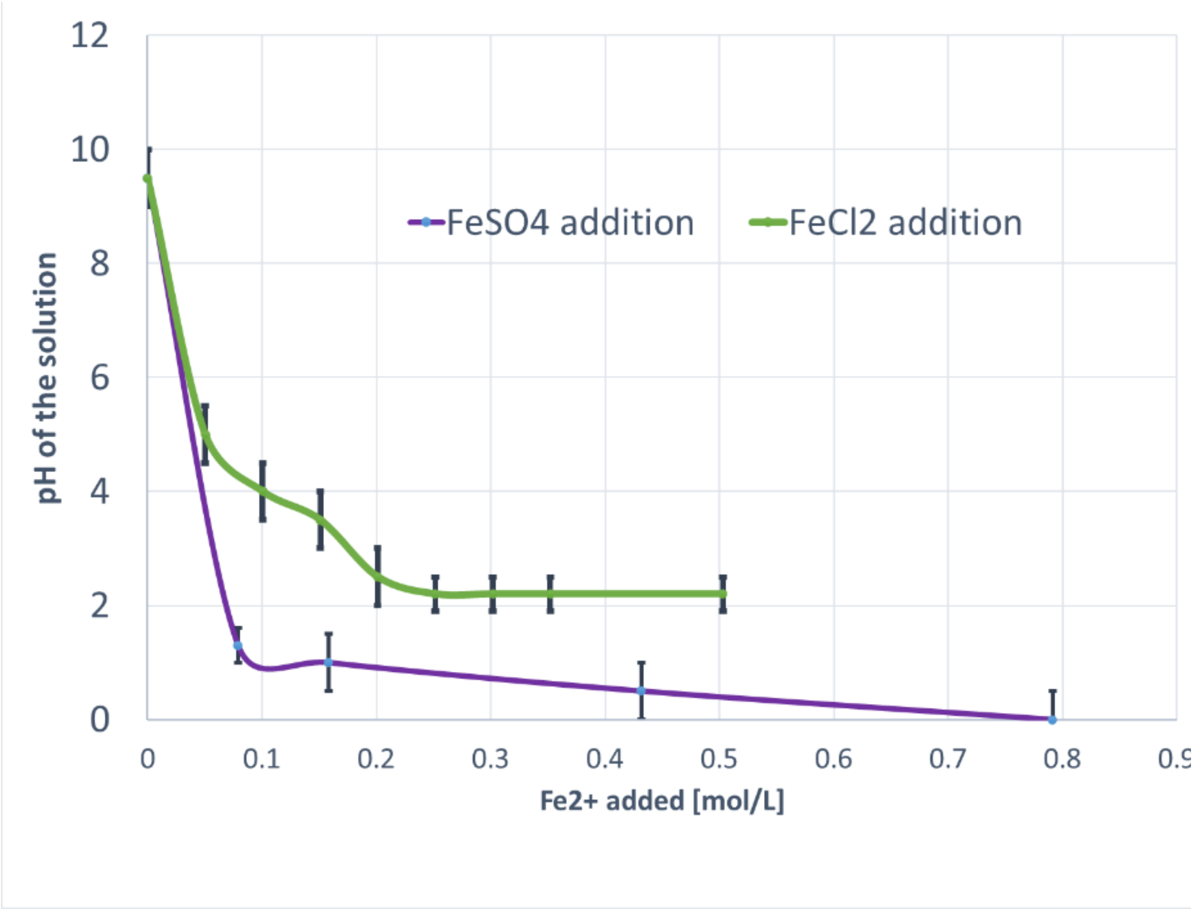

Figure S1: Bentonite slurry pH after addition of Fe(II) salts.

(Green line) pH of the slurry after addition of $FeCl_2.4H_2O$

(Violet line) pH of the slurry after addition of $FeSO_4.7H_2O$

Table S1: Results of titration with $FeCl_2.4H_2O$ with 6.3% bentonite slurry

	Grams of $FeCl_2.4H_2O$ added to 100 ml- 6.3% bentonite slurry	$FeCl_2.4H_2O$ added [converted to mol/L for 100 ml slurry]	pH of the solution after addition of $FeCl_2.4H_2O$	pH error +/-
start solution without $FeCl_2.4H_2O$	0	0	9.5	0.5
added $FeCl_2.4H_2O$	1	0.05	5	0.5
	2	0.10	4	0.5
	3	0.15	3.5	0.5
	4	0.20	2.5	0.5
	5	0.25	2.2	0.3
	6	0.30	2.2	0.3
	7	0.35	2.2	0.3
	10	0.50	2.2	0.3

Table S2: Results of titration with $FeSO_4.7H_2O$ with 6.3% bentonite slurry

	Grams of $FeSO_4.7H_2O$ added to 100 ml-6.3% bentonite slurry	$FeSO_4.7H_2O$ added [converted to mol/L (for 100 ml slurry)]	pH of the solution after addition of $FeSO_4.7H_2O$	pH error +/-
start solution without $FeSO_4.7H_2O$	0	0	9.5	0.5
added $FeSO_4.7H_2O$	2	0.08	1.3	0.3
	4	0.16	1.0	0.5
	12	0.43	0.5	0.5
	22	0.79	0	0.5

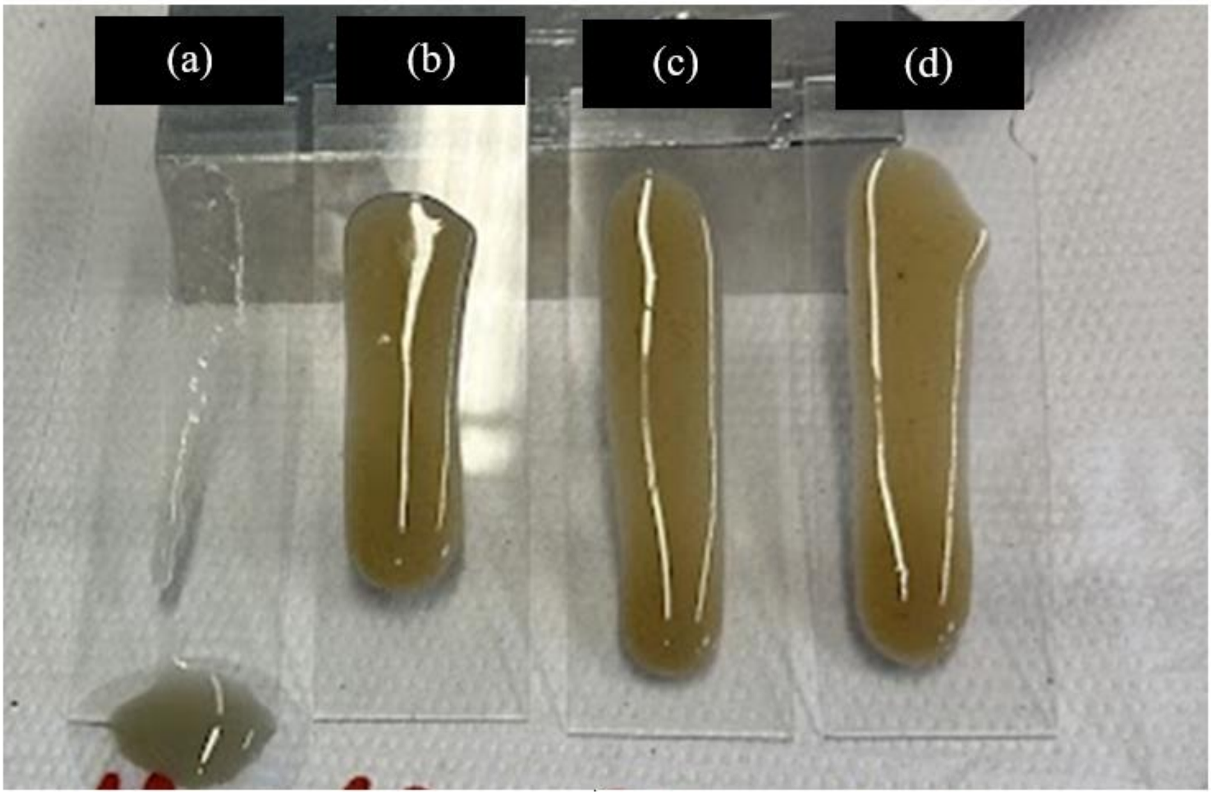

Figure S2: Visual change in the consistency of the slurry after addition of $FeCl_2.4H_2O$.

(**a**) Blank slurry without $FeCl_2.4H_2O$ (seen to be much fluid than the rest of the slurries),

(**b**) 5 g $FeCl_2.4H_2O$ in 100 mL slurry

(**c**) 6 g $FeCl_2.4H_2O$ in 100 mL slurry

(**d**) 7 g $FeCl_2.4H_2O$ in 100 mL slurry

For this test ~1 mL slurry was used as a drop on the tilted glass slides.

Fig. (b, c and d)– are seen to be viscous than the blank slurry.
